# Frequency of bacterial agents isolated from patients with nosocomial infection in teaching hospitals of Mazandaran University of Medical Sciences in 2012

**Published:** 2014

**Authors:** Ali Reza Davoudi, Narges Najafi, Mohsen Hoseini Shirazi, Fatemeh Ahangarkani

**Affiliations:** 1 Antimicrobial Resistance Research Center, Mazandaran University of Medical Sciences, Sari, Iran; 2 Faculty of Medicine, Mazandaran University of Medical Sciences, Sari, Iran

**Keywords:** Nosocomial infection, Bacterial agent, Teaching hospital.

## Abstract

***Background: ***The antibiotic resistance of nosocomial organisms is rapidly increasing. The purpose of this study was to determine the frequency of bacterial agents isolated from patients with nosocomial infection.

***Methods:*** This study was performed in the different wards of teaching hospitals of Mazandaran University of Medical Sciences (northern Iran). The study population consists of the patients with the symptoms of nosocomial infection admitted in these hospitals in 2012. The patient data (including age, sex, type of infection, type of isolated organisms and their antibiotic susceptibility) were collected and analyzed.

***Results:*** The total number of hospitalizations was 57122 and the number of nosocomial infection was 592. The overall prevalence of nosocomial infection was 1.03% that was mostly in Burn unit and intensive care unit. The most common nosocomial infection was wound infection (44.6%) and the most common organisms were Pseudomonas aeruginosa and Acinetobacter.

***Conclusion: ***Given the increasing numbers of nosocomial infection in this region, especially infection with Pseudomonas aeruginosa, it is necessary to make a precise reporting and improve the procedures of infection control in hospitals.

Despite tremendous advances in the treatment of human infectious diseases over the 20th century, the problem of hospital-acquired infection remains a critical issue, not only its low incidence, but its importance is increasing day by day. Globally, 8.7% of hospitalized patients had nosocomial infections. These infections cause the increasing rate of deaths, failure of surgeries, rejection of organ transplantation, failure of chemotherapies and increasing patient cost sharing, likewise in health centers, a longer hospital stay causes mental and emotional stress (1). Urinary tract infection is the most common nosocomial infection in the world (about 40 %) and the most important risk factor of this infection is urine catheter insertion. The main etiologic agents are gram negative bacilli including Ecoli, Klebsiella Pneumoniae and Pseudomonas Aeruginosa (2). The rate of nosocomial pneumonia is estimated at 5-10 episodes per 1000 hospitalizations. The rate of ventilator associated pneumonia (VAP) is related to the length of the mechanical ventilation. Important etiological factors are gram negative bacilli such as Pseudomonas aeruginosa, Klebsiella pneumoniae, Acinetobacter species and Staphylococci (3).

The rate of bacteremia associated with intravascular devices increased significantly these years. 19% of bacteremia were attributed to the catheter- related infection. Surgical site infections (SSI) are the most common in patients after surgery comprising 14-16 percent of all such infections. Recognizing microbial etiology and antibiotic resistance pattern is vital in any region, hospital epidemiologic plans and antibiotic susceptibility patterns in hospitals should be regularly monitored (6). The purpose of this study was to determine the frequency of nosocomial infections and antibiotic susceptibility patterns of teaching hospitals affiliated to Mazandaran University of Medical Sciences to help the physicians in choosing better antibiotics for initial empiric therapy. 

**Methodology:** This is a descriptive cross sectional-retrospective study. The location of the study in different wards of teaching hospitals of Mazandaran University of Medical Sciences (northern Iran) included Imam Khomeini, Fateme Zahra, Shahid Zare, BuoAli Sina and Razi hospitals. The study population comprised of the patients hospitalized in these hospitals in 2012 who had symptoms of nosocomial infections. The most common types of infections (based on the National Directory of Nosocomial Infections Surveillance System, NNIS) (7) are wound infection, blood infection (bacteremia), urinary tract infection and respiratory infections, defined as follows:

**Urinary Tract Infection:** The patient must have at least one of these symptoms such as fever, dysuria, frequency, flunk pain, suprapubic pain, nausea and vomiting plus positive urine culture or at least must have two symptoms such as fever, dysuria, frequency, flunk pain, suprapubic pain, nausea and vomiting plus pyuria.

**Wound Infection:** Superficial surgical site infection is identified with at least one of the following characteristics: purulent discharge from the wound, organisms isolated from the fluid or superficial surgical tissue that is prepared aseptic, at least one of the symptoms like pain, swelling, redness or hot, or diagnosis of the wound infection by the doctor.

**Respiratory Infection:** Hearing the crackles on lung examination or radiographic findings plus at least one of the following, characteristics: purulent sputum or positive blood culture or positive culture of tracheal aspirate sample.

**Blood Infection:** Blood culture growing a pathogenic organism, a condition that is not related to the location of a localized infection or having fever, chills, lowering the blood pressure level plus existing infections related to the skin in at least two blood cultures (like diphteroids, bacillus species, propionibacterium or coagulase negative staph).

In the beginning, we provided a questionnaire that included the demographic and clinical characteristics, risk factors, medical history, main diagnosis, type of nosocomial infection and sort of culture. The antibiotic susceptibility was determined by the Kirby Bauer Disk. Then, we took out the list of patients with nosocomial infection that was provided by the hospital infection control nurse and then we went to the archive file of the patients one by one. We extracted our required patient data and entered them in the information form. Finally, the collected data were analyzed using the SPSS Version 13 (including the descriptive statistics with median values).

## Results

**Figure1:**
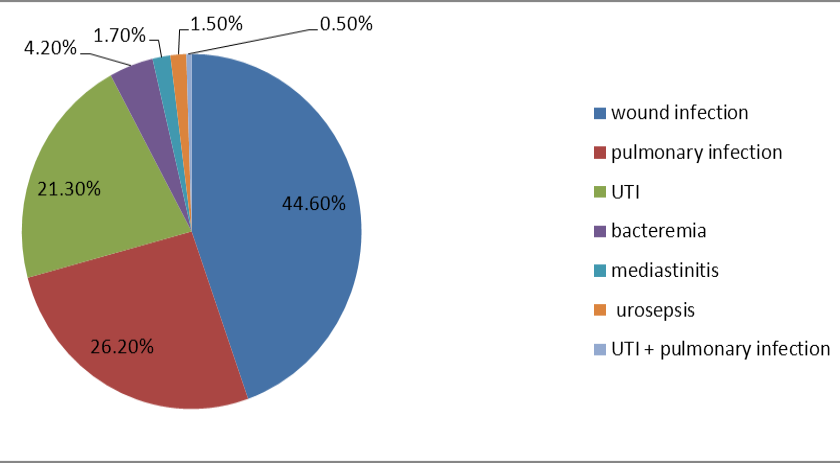
Frequency of nosocomial infections in patients hospitalized in teaching hospitals of Mazandaran University of Medical sciences in 2012

**Figure 2:**
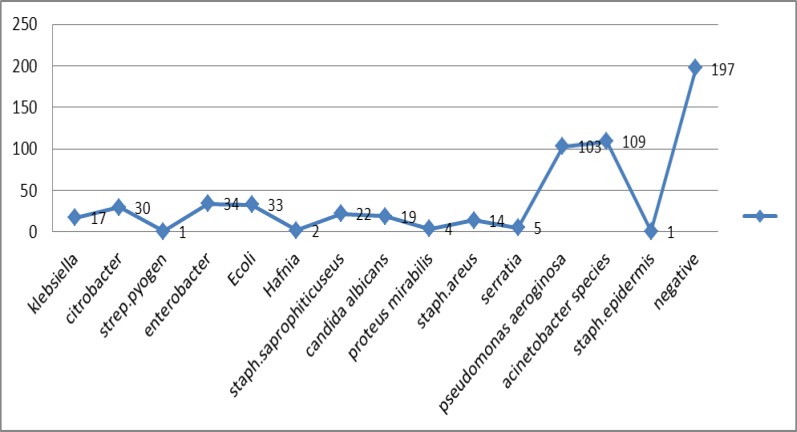
Frequency of organisms taken out from patients infected with nosocomial infection in teaching hospitals of Mazandaran University of Medical sciences in 2012

The total number of hospitalizations of our hospitals was 57122 patients and the number of nosocomial infection was 592. Of these patients, 303 were males (51.2%) and 289 were females (48.8%). The average age was 52±22.5 (range, 16-91) years. The average duration of hospitalization was 7.4 (range, 1-91) days. The total prevalence of nosocomial infection was 1.03% that most of it was seen in the Burn unit and intensive care unit ICU, respectively. Wound infection was the most common nosocomial infection in 264 patients (44.6%) which was shown in figure 1. The most common organisms were Pseudomonas aeruginosa and Acinetobacter (109 and 103 cases, respectively) (figure 2).

The clinical characteristics, etiologic agents and the risk factors of every infection were described: 

**Wound infection:** Totally, 264 patients had wound infection 135 men, (51.1%) and 129 women (48.9%). The average age was 47.6±21.3 years old. The most common sign was wound erythema in 251 (95%) patients. The other signs were wound oozing (81%), and suture openings (63.3%). The most common risk factors for this infection were respectively diabetes mellitus (35.6%), HTN (31.4%), cardiovascular diseases (25.3%) and those using steroids (23.1%). The most common germs were Staphylococcus aureus (43.8%), Pseudomonas aeruginosa (24.2%), Acinetobacter (21.8%), Citrobacter (8.9%) and Entrobacter (7%). 

**Respiratory Infection:** Totally, 155 patients were infected with nosocomial pneumonia (52.2% males and 47.7% females). The average age was 59.1±22.6 years. The most common symptoms were fever (100%), cough (89.6%), increase of sputum (92.2%) and dyspnea (76.7%), respectively. The most common risk factors were intubation, mechanical ventilation (68.9%), and then, cardiovascular disease (31.6%). The most common organism was Acinetobacter (seen in 18.8% of patients). The other organisms were Pseudomonas aeruginosa (16.2%), Klebsiella Pneumoniae (7%), Staphylococcus aureus (6.5%), Entrobacter (5.1%) and Citrobacter (1.2%).

**Urinary tract infection:** Nosocomial UTI was seen in126 patients (43.7% males and 55.6% females). The average age was 54.1±21.8 years. The most common symptoms were fever (98.4%), dysuria (87.4%), frequency (77.7%) and flunk pain (31.7%). The most common risk factor was catheterization (73.6%). The most common organisms were E.coli (32%), candida (19.3%), Acinetobacter (17%), Pseudomonas (11.7%) and Entrobacter (10.6%), respectively.

**Bacteremia:** Blood infection developed in 25 patients (68% were males and 32% females). The average age was 46.3±28.2 years, and the most common symptom was fever in all patients. The most risk factor for cardiovascular disease was 56% in patients. The most abundant organisms taken out were Staphylococcus epidermis (31%), Entrobacter (16%).

## Discussion

The highest rate of nosocomial infection in this study was seen in the Burn unit and then in the ICUs. The burned patients in the acute phase, almost died because of vital organ failure and in the patients that passed the acute phase, infection is its most reason (8). In Taylor et al’s study, the rate of nosocomial infection in the Burn unit was reported 32.8 per 1000 hospitalization (9). Because of the nature of the burned tissue, suppressive effects of burns, length of hospital stay and manipulating during admission (such as catheterization, intubation, blood collection, etc.), these patients are at high risk of nosocomial infection (9, 10). From our 592 patients, 101 cases (17.1 %) were admitted to the ICU. The rate of nosocomial infections in ICUs is nearly 20%. The ICU patients are prone to infections like bacteremia (occur almost because of intravascular instruments), pneumonia (VAPs), UTIs (because of using of urinary catheter) (11). The prevalence of these infections in the ICU patients is 5 -10 times higher than the general wards. In Sohrabi's study, the rate of infection in ICUs was reported to be very high and it was 54.1 per 10000 patients per day (12). The average age of our cases was 52.2±22.5. In Sohrabi's study, the average age was 55.7±23.9 that was similar to our study (12). The average of over 50 years old can demonstrate older people with a greater risk of infections (13). Also, it was specified that people over 50 are the most resistant antibiotics (14). In our study, E.Coli isolated from 30 patients (23.8%) was the most common cause of UTI and Acinetobacter from 16 cases (12.7%) was the second cause. Although Pseudomonas aeruginosa and Acinetobacter are the most common cause of nosocomial infections in the ICUs and are resistant to a wide range of antibiotics, but, in many studies on nosocomial infections the main cause of UTI is still E. Coli (15). The most common risk factor for UTI was urine catheter insertion (73.8% of patients). This was reported in many other studies, too (16). In respiratory infections, the most common pathogen was Acinetobacter (in 29% of patients). Acinetobacter species are the most common gram negative bacteria in nosocomial infection. These germs are resistant to a wide range of antibiotics. Hashemian studied about antibiotic therapies in patients with VAPs in Masih Daneshvari Hospital. In his study, the most common germ was Acinetobacter, too (17). In our study, the second common microorganism that was isolated in culture was Pseudomonas aeruginosa (103 patients, 17%). Zadegan et al. studied about the rate of Pseudomonas aeruginosa infection in hospitalized patients in Baghiatallah Hospital. In that study, the most common germ in bronchial samples was Pseudomonas (33.5%) (18). In another study, the prevalence of Pseudomonas aeruginosa in nosocomial infections was reported to be 20% (19). These differences in the prevalence of Pseudomonas could be due to hospital environment and health (20). In our patients, 221 cases (37.3%) were diabetic, so diabetes seems to be a major risk factor. In a study on 2005 in an orthopedic ward, diabetes was one of the predisposing factors for nosocomial infections (16). The increase use of Foley catheter, duration of diabetes, long stay in hospital and diabetic neuropathy are the reasons for the increase of nosocomial infection risks in diabetic patients (21).

This study has limitation based on the study design which was retrospective and so the susceptibility test was limited to some antibiotics of isolated organisms.

In conclusion given the increasing number of nosocomial infection in our region, it is necessary to make precise reporting and improving procedures of infection control in hospitals.

## Notes

**Funding:** This project was supported by a grant received from the Vice-Chancellery for Research of Mazandaran University of Medical Sciences.
